# Advancements in Cyclodextrin Complexes with Bioactive Secondary Metabolites and Their Pharmaceutical Applications

**DOI:** 10.3390/pharmaceutics17040506

**Published:** 2025-04-11

**Authors:** Oana Elena Nicolaescu, Cătălina Ionescu, Adriana Samide, Cristian Tigae, Cezar Ionuţ Spînu, Bogdan Oprea

**Affiliations:** 1 Department of Pharmaceutical Technique, Faculty of Pharmacy, University of Medicine and Pharmacy of Craiova, 2 Petru Rareş, 200349 Craiova, Dolj, Romania; oana.nicolaescu@umfcv.ro; 2 Department of Chemistry, Faculty of Sciences, University of Craiova, 107i Calea București, 200144 Craiova, Dolj, Romania; adriana.samide@edu.ucv.ro (A.S.); cristian.tigae@edu.ucv.ro (C.T.); cezar.spinu@edu.ucv.ro (C.I.S.); 3 Faculty of Medicine, University of Medicine and Pharmacy of Craiova, 2 Petru Rares, 200349 Craiova, Dolj, Romania; bogdan.oprea@umfcv.ro

**Keywords:** cyclodextrins, natural compounds, secondary metabolites, polyphenols, terpenes and terpenoids, alkaloids

## Abstract

Cyclodextrins (CDs) have largely been investigated during the last decades for their outstanding properties, such as biocompatibility and biodegradability, with wide applications in the pharmaceutical field, among which the formation of inclusion complexes (ICs) with natural or synthetic lipophilic compounds. This review prioritizes the research of recent years (2022–2025), being focused on (1) systematization of the research of ICs based on the structure of the secondary metabolite, namely (i) polyphenols (PPs), (ii) terpenes and terpenoids (TTs), and (iii) alkaloids (Alks); (2) for each type of inclusion complex, the following aspects have been discussed: benefits of complexation, composite materials, and in vitro/in vivo and theoretical studies; and (3) pharmacokinetics and pharmacodynamics, risks, limitations, and perspectives of cyclodextrin inclusion complexes with secondary metabolites.

## 1. Introduction

Human and veterinary pharmaceutical compounds represent a source of chemical substances with high environmental impact. The manufacturing process of pharmaceutical compounds contributes to air pollution mainly by using halogenated compounds, impacting air quality and human health. Pharmaceutical compounds are residual chemicals in wastewater; they pollute soils and groundwater, and their toxicity affects aquatic and terrestrial ecosystems and human health [1,2]. Therefore, due to the wide applications, incomplete removal and persistence of pharmaceutical substances in the environment, the research of new alternatives is of highest importance. Lately, much interest has been devoted to the study of non-toxic, biodegradable compounds able to express pharmacological activity. Natural compounds are an excellent resource, but their application is often limited because of their low water solubility. A solution to this problem is constituted by employing cyclodextrins (CDs) as encapsulation agents for biologically active substances with low solubility [3]. CDs are obtained using non-polluting methods and are biodegradable. Therefore, the inclusion of CD complexes with natural compounds offers a double advancement in the development of the green pharmaceutical concept.

CDs are cyclic oligosaccharides made from α-D-glucopyranoside units linked by α-1,4-glycosidic bonds, formed from starch through degradation under the enzymatic action of cyclomaltodextrin glucanotransferase (E.C. 2.4.1.19; CGTase) [4,5]. They are crystalline, non-reducing, and hydrophilic, and they have the property of forming inclusion complexes (ICs) with hydrophobic compounds, favoring their water solubilization. The extensive research devoted to CDs focuses on their ability to form ICs with molecules that fit within their internal cavity and on the study of their applications.

The first reference to CDs dates from 1891, when the French pharmacist and chemist Antoine Villiers mentioned a substance that later turned out to be a cyclodextrin [6]. By studying the breakdown products of potato starch, produced enzymatically under the action of the bacterium *Bacillus amylobacter*, A. Villiers discovered crystalline dextrins (cellulosines). Since then, researchers’ interest in this subject has undergone continuous growth. Now, 52,608 articles and 3519 reviews with “cyclodextrin” as a topic are being indexed in the Web of Science Core Collection (accession date: 12 January 2025). Some of these papers review the history [7,8], structure [9,10], and industrial applications [11,12] of CDs. The main applications can be found in fields such as the food industry, dietary supplements, the pharmaceutical and cosmetic industries, agriculture, environmental protection and wastewater treatment, and the textile industry.

The pharmaceutical applications of CDs involve the synthesis of inclusion complexes (ICs) formed by CDs with various lipophilic drugs. An important part of the bioactive compounds consists of natural compounds, secondary metabolites with various activities, such as antihyperglycemic, anti-inflammatory, analgesic, anticoagulant, antihypertensive, antioxidant [13], antimicrobial [13,14,15], antitumor [16,17,18], antidepressant, antitussive, antidiarrheal, sedative and hypnotic, and antimalarial activities [19,20]. In the early 1900s, before synthetic drugs began to be produced industrially in large quantities, 80% of medicines were derived from plant-based sources, such as extracts from leaves, roots, and bark [21].

The notion of a “natural product-derived drug” is debatable, but it is estimated that around 50% of drugs are of natural origin [22]. For some types of treatments, the percentage is even higher; for example, 60% of anticancer agents and 75% of anti-infectious agents are considered to be natural or naturally derived compounds [21].

There are numerous other bioactive natural compounds that have not been discovered, as it is estimated that only around 10% of the world’s natural resources have been explored so far [23]. The land has been far more explored than the ocean, as the ocean is not our natural habitat. Until the 1970s, people did not even consider investigating the ocean [23,24], and even today, a vast number of marine species remain unexplored.

CDs also have other applications in related fields, such as biotechnology and analytical chemistry. Among these applications, the following can be found: (a) the use of CDs in plant tissue and organ culture for the biotechnological production of certain plant SMs by elicitation [25,26,27] and (b) the employment of CDs as stationary phase for enantioselective separations in the purification of secondary metabolites through column chromatography [28,29].

**Figure 1:**
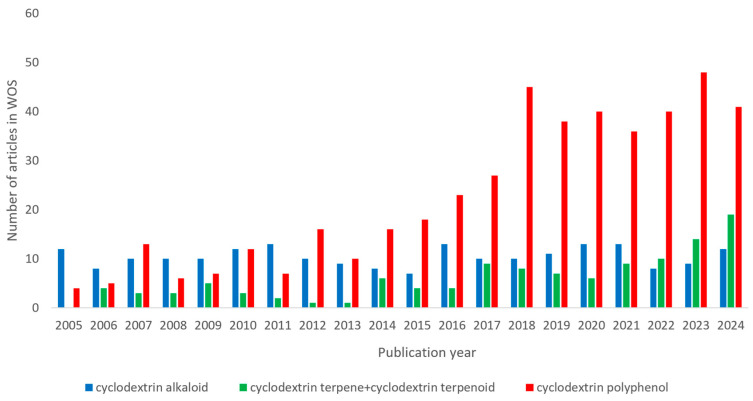
Trends in the number of articles indexed in the WOS Core Collection over the last two decades. Note that the data were obtained by searching for articles with the following keywords: blue bars—“cyclodextrin” and “alkaloid”; green bars—“cyclodextrin”, “terpene” + “cyclodextrin”, and “terpenoid”; red bars—“cyclodextrin” and “polyphenol” (collection date: 10 January 2025). The vertical bars represent the number of articles indexed in the WOS Core Collection for each of the keywords detailed above and reflect the dynamics of research on each type of inclusion complex discussed in this review.

In the last 20 years, a considerable amount of research has focused on the interface between CDs and secondary metabolites. As shown in Figure 1, a large number of articles indexed in the Web of Science (WOS) Core Collection address cyclodextrins and specific types of secondary metabolites, such as polyphenols (PPs), terpenes and terpenoids (TTs), and alkaloids (Alks).

In Figure 1, the following trends can be observed:

(a) Two decades ago (years 2005–2014), the number of articles on the topics of CDs and secondary metabolites was quite stable, with a predominance of studies on CD-PPs over CD-Alks, with the least interest in CD-TTs;
(b) In the last decade (years 2015–2024), different trends were observed for various classes of secondary metabolites: (i) the number of articles on CD-PPs showed a progressively increasing interest, with a mean of around 40 articles/year over the last 7 years; (ii) the number of articles on CD-TTs remained relatively stagnant in the first half of the last decade, followed by a steady increase in the last 5 years, with a mean of around 10 articles/year in that period; and (iii) the number of articles on CD-Alks remained constant throughout the last decade, with a mean of around 10 articles/year over the last 10 years.

Overall, a predominance of interest in CD-PP complexes may be observed. The flow of publications is dynamic, and the data collected in Figure 1 are valid until 10 January 2025. In the context of the growing number of articles on this topic, the synthesis and applications of CD complexes with natural compounds in various domains have been reviewed [30,31,32].

The current review proposes an update on recent advances in the research of inclusion complexes (ICs) formed between CDs and SMs with pharmaceutical applications. A systematization of the complexes obtained in the last three years, based on the structure of the complexed SMs, was achieved. The main classes reviewed include polyphenols (PPs), terpenes and terpenoids (TTs), and alkaloids (Alks). Accordingly, inclusion complexes (ICs) of cyclodextrins (CDs) with the previously mentioned secondary metabolites (PPs, TTs, and Alks) will hereafter be referred to as follows: ICs of CDs-PPs, ICs of CDs-TTs, and ICs of CDs-Alks.

## 2. Structure, Properties, and Main Applications of Cyclodextrins

### 2.1. Structure of Cyclodextrins

**Figure 2:**
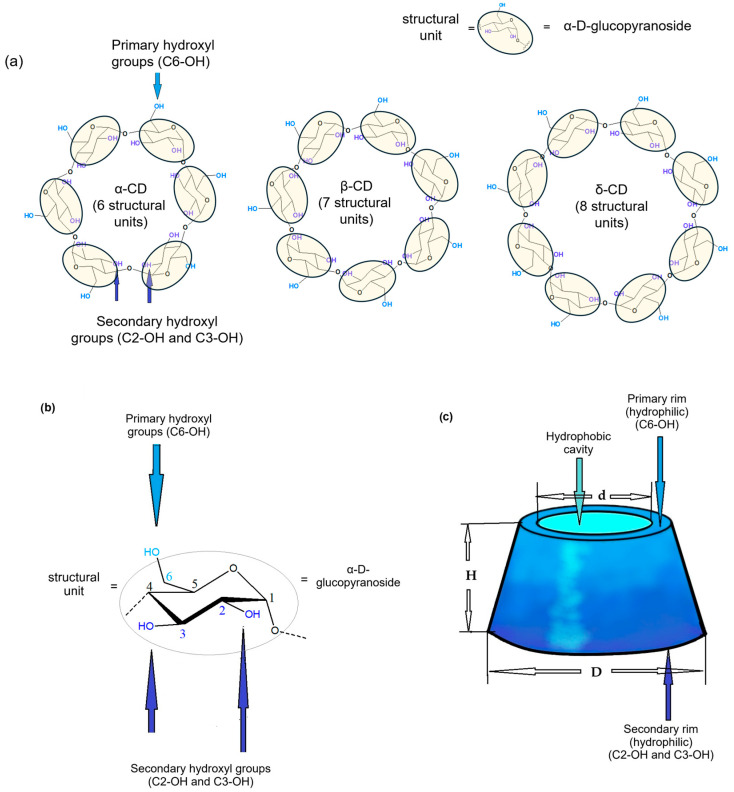
Representations of CDs: (**a**) chemical structure of α-, β-, and δ-CDs; the number of structural units (α-D-glucopyranoside) is six (for α-CD), seven (for β-CD), or eight (for δ-CDs); (**b**) chemical structure of α-D-glucopyranoside unit; primary (C6-OH; blue color) and secondary (C2-OH and C3-OH; violet color) hydroxyl groups are indicated; numbers 1–6 indicate C1–C6 atoms of α-D-glucopyranoside unit; (**c**) schematic representation of the three-dimensional shape of α-,β-, and δ-CDs, highlighting the hydrophobic and hydrophilic properties of β-CD; meaning of d, D and H notations.

Figure 2 shows the two- and three-dimensional structures of natural CDs that have been previously described in the literature [10,33,34,35,36]. There are three types of native (natural) CDs used in practice, with the difference between them being the number of α-D-glucopyranoside structural units present in their macrocycles: α-CDs (six structural units), β-CDs (seven structural units), and δ-CDs (eight structural units) (Figure 2a,b). CDs have a known three-dimensional shape of a toroid (truncated cone) [37], illustrated in Figure 2c, and are composed of two rims (wide and narrow) and an internal cavity [10,33,35,38], which define their hydrophilic/hydrophobic properties.

The cyclodextrins α-, β-, and δ-CDs have the same height (H) (7.9 Å), but their cavity dimensions increase. The internal diameter (d) varies between 4.5–5.3 Å and 6.0–6.5 Å for α- and β-CDs and from 7.5 to 8.3 Å for δ-CD, respectively, while the external diameter (D) at the broad rim reaches the values of 14.6 Å and 15.4 Å for α- and β-CDs and 17.5 Å for δ-CDs, respectively [39,40]. The graphical representation of the height (H), internal (or inner) diameter (d), and external diameter at the broad rim (or outer diameter) (D) is displayed in Figure 2c. As the number of carbohydrate cycles increases, cavity volume increases, too [33,41].

### 2.2. Properties of Cyclodextrins

#### 2.2.1. Hydrophilic/Hydrophobic Properties of Cyclodextrins

According to the literature data [10,33,35,38], the structure shown in Figure 2c exhibits the following characteristics: (a) two polar edges responsible for the hydrophilic behavior of CDs namely, a wide rim (“secondary face”), where the secondary hydroxyl (-OH) groups are attached to the carbon atoms (C2, C3) and a narrow rim (primary face), where the primary -OH groups are bonded to C6 carbon atoms; (b) an internal cavity, where carbohydrate ring atoms and glycosidic oxygen atoms are present, inducing the hydrophobic properties [10,33,35,38].

Therefore, CDs are suitable for the incorporation of hydrophobic molecules into the inner cavity through host–guest interactions, replacing the weakly bound water molecules. CDs represent the hosts, while the incorporated substances are the guests, thus forming the water-soluble inclusion complexes that can favor the solubilization of hydrophobic compounds.

#### 2.2.2. Solubility of Cyclodextrins

CDs are insoluble in most organic solvents, but they are water-soluble. The water solubility of CDs increases proportionally with temperature and the number of carbohydrate units [33], but abnormally low solubility was recorded for β-CDs. This can be explained by taking into consideration the stereochemistry of hydroxyl groups (-OH) at the C2 and C3 carbon atoms [42]. In β-CDs, these groups are oriented towards each other, thus forming a strong connection between them; therefore, they interact to a lesser extent with water molecules compared to the C2 and C3 hydroxyl groups of α- and δ-CDs.

#### 2.2.3. Toxicity of Cyclodextrins

Chemically modified CDs possess different toxicities when compared to their native ones. It is generally accepted that native CDs have virtually no toxicity when administered via the oral route, as they are not absorbed. When administered parenterally, there is a different toxic effect, as CDs enter the systemic circulation. β-CD cannot be administered parenterally due to its gastrointestinal and renal toxicity [43,44,45]. When modified (by methylation, for example), their lipophilicity increases and approximately 10% of the quantity is absorbed in the gastrointestinal tract. Therefore, limitations exist regarding the quantity administered orally; additionally, CDs cannot be administered via the parenteral route [43,44]. A derivative of β-CD, 2-hydroxypropyl-β-cyclodextrin (HP-β-CD), has cholesterol-chelating properties that disrupt cellular membranes, and its usage has been linked to hearing loss in various animal species (rats, cats), as well as in humans [46,47,48,49]. Inclusion complexes (ICs) of cyclodextrins (CDs) with various drugs may also induce drug-related toxicity due to increased concentrations of the released drugs above the therapeutic threshold [50].

#### 2.2.4. Biocompatible and Biodegradable Properties of Cyclodextrins

The performance of cyclodextrin hosts to modify the chemical and biological properties of incorporated molecules (guests) leads to the development of systems that are easily tolerated by the human body, reflecting good biocompatibility [33]. Also, cyclodextrins do not cause major side effects such as allergenic or cytotoxic reactions.

Cyclodextrins accumulate in the body in small amounts, as they are biodegradable compounds. Test results revealed that β-CDs were almost completely eliminated from the plasma of rats within 36 h. It is assumed that the main metabolic product is maltodextrin, which is absorbed and subsequently metabolized and eliminated in the form of carbon dioxide and water [51].

### 2.3. Applications of Cyclodextrins

#### 2.3.1. Application Fields of Cyclodextrins

Cyclodextrins (CD_S_) have applications in a wide range of domains, including various branches of the chemical industry, such as agrochemistry, supramolecular chemistry and chromatography, enzymology and catalysis, but also in related fields, such as cosmetics, food science, the textile industry, biology, medicine, and pharmacy [52,53,54,55]. Cyclodextrins are important tools in the pharmaceutical industry for nutraceutical production, with their main application being the obtention of natural products that have increased antioxidant properties. CDs have also been reported as green and safe additives that improve the characteristics of the leather tanning process through interactions between CD-derived composites and collagen, the main protein in skin [56,57]. These findings may have applications in the future for the sustainable industrial processes of leather tanning.

#### 2.3.2. Pharmaceutical Applications of Cyclodextrins

Cyclodextrins are biocompatible, biodegradable, and non-carcinogenic compounds with low toxicity and high stability. They are tasteless, colorless, and odorless, properties that make them perfect candidates for drug encapsulation [33,58]. CDs form inclusion complexes with lipophilic drugs, making them water-soluble. The main benefits of the implementation of CDs in the pharmaceutical field consist of (a) increasing drug solubility and stability (photo- and thermostability), (b) reducing the drug dose for the same efficacy, (c) masking the unpleasant taste or smell of drugs, (d) decreasing drug side effects, and (e) controlling drug release [43,59,60,61,62].

The most studied natural cyclodextrin (CD) for pharmaceutical applications is β-CD, as it possesses a cavity volume suitable for the encapsulation of most drugs, but its water solubility is inferior to that of α- and β-CDs. However, the solubility of native CDs may be altered through chemical modifications: (a) methylation—increases lipophilicity and the capacity to encapsulate hydrophobic compounds; (b) hydroxypropylation—increases hydrophilicity and water solubility; (c) sulfation—increases hydrophilicity; (d) phosphorylation—increases hydrophilicity; and (e) acylation—increases water solubility [60,62].

## 3. Inclusion Complexes of Cyclodextrins

### 3.1. Formation Mechanism of Inclusion Complexes of Cyclodextrins

As previously specified, the wide and narrow rims of cyclodextrins have hydrophilic properties, while their inner cavities are hydrophobic, acting as a host for different guests, thus leading to the formation of cyclodextrin inclusion complexes. The guest replaces the pre-existing water molecules in the cyclodextrin cavity and subsequently binds to it, most likely through intermolecular hydrogen bonds [63,64,65,66].

Also, other non-covalent bonds, namely van der Waals, electrostatic, or hydrophobic bonds, can also be involved in cyclodextrin-guest interactions [67]. The complexation efficiency depends on the properties of the guest, which also influence the stability of inclusion complexes.

Consequently, cyclodextrins (hosts) act as protective coatings for certain incorporated substances (guests), making them suitable for the encapsulation of pharmaceutical products and/or some primary/secondary metabolites. Coordinate covalent (dative) chemical bonds between the host and guest could be formed if there are suitable functional groups in the secondary metabolite molecule that are capable of donating electrons to the vacant orbitals of the atoms in the cyclodextrin macrocycle.

### 3.2. Preparation Methods of Inclusion Complexes of Cyclodextrins

One of the most well-known methods for preparing cyclodextrin inclusion complexes is coprecipitation, which is applied to water-insoluble substances. Primarily, the guest is dissolved in an organic solvent, generally ethyl alcohol, which is then mixed under stirring with a cyclodextrin aqueous solution [67].

Grinding is an ecological mechanical method that does not require solvents and involves shattering the crystals to increase the contact surface between the host and the guest, thus intensifying the interactions between cyclodextrins and the guest compounds. Grinding is an efficient and economical technique that is suitable, especially for the pharmaceutical industry [67,68].

The paste method, or kneading, consists of preparing a paste from accurately weighed cyclodextrin and water, into which the guest substance is incorporated under continuous mixing [65,69].

Other conventional methods to prepare cyclodextrin inclusion complexes, such as freeze drying (lyophilization) [70], spray drying, and synthesis under microwave irradiation or in supercritical carbon dioxide, have also been reported [67].

### 3.3. Confirmation and Analysis of Inclusion Complexes of Cyclodextrins

Fourier transform infrared spectroscopy (FTIR) is a widely used method for the analysis of chemical compounds. It is also applied to confirm the formation of inclusion complexes, e.g., those of β-CD [63,64,70,71,72] and its derivative HP-β-CD [69,71,72] with certain guests, including quercetin [63,72], epigallocatechin-3-gallate (EGCG) [64], chrysin [69], luteolin [70], curcumin [71], and resveratrol [72].

Scanning electron microscopy (SEM) images reveal changes in the surface morphology of the inclusion complex compared to that of cyclodextrin. The SEM images illustrate specific surface characteristics for some inclusion complexes of β-CD and HP-β-CD, such as β-CD-curcumin and HP-β-CD-curcumin [71]; HP-β-CD-chrysin [69]; β-CD-resveratrol [68]; β-CD-catechin, β-CD-gallic acid, and β-CD-epigallocatechin gallate [73]; β-CD-curcumin [74]; and HP-β-CD-ellagic acid [75].

UV-Vis spectrophotometry, in some cases, reveals a spectrum for the inclusion complex that differs from the original spectrum of the guest. Β-CD-luteolin [70] and α-, β-CD-hydroxytyrosol [76], prepared using the freeze-drying method, were analyzed using UV-Vis spectrophotometry.

Simultaneously, thermogravimetric/differential scanning calorimetry (TG/DSC) was also applied to obtain information on the mass change within a temperature range, accompanied by the specific endothermic/exothermic thermal effects for certain inclusion complexes compared to the host. The inclusion complexes, such as β-CD-curcumin and HP-β-CD-curcumin [71], γ-CD-phloroglucinol [77], and HP-β-CD-ellagic acid [75], were investigated using TG/DSC thermal analysis. Additionally, on the TG curve, additional peaks related to the melting points of chemical substances can be highlighted. In this case, the process proceeds without mass variation, and therefore, no endo/exo peaks are recorded on the heat flow diagram.

Dynamic Light Scattering (DLS) allowed for the determination of size, polydispersity index, and ζ-potential values for the obtained ICs. DLS has been successfully applied to characterize complexes such as HP-β-CD-resveratrol and HP-β-CD-quercetin [72], but also various composites containing ICs of β-CD, HP-β-CD, and methyl-β-CD with polyphenol-rich extracts.

Other techniques such as X-ray diffraction (XRD) [63,64,65,68,70,74,77], nuclear magnetic resonance (NMR) [63,69,76,78,79,80,81], high-performance liquid chromatography (HPLC) [65,72,79,82,83,84,85,86], and electrospray ionization tandem mass spectrometry (ESI-MS/MS) [73] were successfully applied to confirm some inclusion complexes.

The analysis methods of ICs must be judiciously chosen to accurately confirm the formation of CD-SM complexes. Each analysis method provides valuable information regarding complexation but also has specific limitations, as detailed below. SEM images display the surface morphology and can provide information about the similarities/differences between the morphology of the inclusion complex and that of the cyclodextrin host. In contrast, they do not provide any clues about the interactions leading to the formation of the inclusion complex [67]. UV-Vis spectrometry is a rapid technique that requires small amounts of substances. The UV-Vis spectrum shows modified characteristics compared to the initial spectrum of the secondary metabolite. In the case of the compound formulated with cyclodextrins, the spectrum generally shows alterations in wavelength and baseline, along with a weakly perceptible absorbance maximum. In conclusion, UV-Vis spectrophotometry can indicate changes in the initial composition but cannot precisely specify the causes that generated these changes. Thermal analysis is performed over a limited time and provides information on the steps taken during thermal decomposition, as well as the endothermic or exothermic processes that accompany them. The thermoanalytical curves recorded for inclusion complexes should be analyzed in comparison with those of cyclodextrin and the secondary metabolite, as their decomposition may involve overlapping processes characteristic of the thermal decomposition of CDs or/and SMs. Additionally, extra steps observed during the analysis of complexes can be easily confused with a simple treatment applied to the metabolite and not necessarily the complex formation. FTIR is a widely used technique that can be performed in a limited time and is relatively inexpensive. Information about the positioning of bonds within complexes can be obtained; however, caution is required during sample preparation [67]. NMR is one of the most efficient techniques for studying host–guest interactions. It provides information about the orientation of the secondary metabolite within the host cavity, but formation constants obtained in deuterated solvents may differ slightly from those measured in water [67].

Consequently, to ensure accurate confirmation of inclusion complexes, multiple analytical methods should be used in combination.

### 3.4. Guests of Cyclodextrins in This Review: The Secondary Metabolites

In 1981, Albrecht Kossel proposed the differentiation of metabolites into two categories: primary and secondary [87]. The term remained in use, later being taken over by Friedrich Czapek in his book *Plant Physiology* and has been used since then [88]. The term “natural products” is also used for “secondary metabolites” by chemists in order to distinguish them from compounds that typically fall within the study domain of biochemists. Unlike primary metabolites, which have a ubiquitous presence in nature, secondary metabolites (SMs) are restricted in occurrence, being predominantly expressed in certain organisms, sometimes within a single family, genus, or even species, and they have a unique presence in specific plant organs or tissues [89]. It is generally accepted that primary metabolites play roles in the growth and development of organisms and are essential for life. Examples of primary metabolites include biomolecules such as proteins, carbohydrates, lipids, and nucleic acids, which are being studied by biochemists. To clarify, secondary metabolites do not play a role in the growth and development of organisms and are not essential for life [90]. Instead, they play a role in “chemical ecology”, modulating intra- and interspecific interactions among living organisms.

There is a blurry line of distinction between primary and secondary metabolites. Moreover, a high number of secondary metabolites exist, exhibiting impressive structural diversity, which has led to various classifications within the category of SMs [91,92,93,94]. Three main classes of secondary metabolites can be distinguished as follows:

(a) Phenolic compounds, most of them being polyphenols (PPs) [95];
(b) Terpenes and their oxygenated derivatives, terpenoids (TTs); other isoprene-derived compounds, such as steroids, carotenoids, and gibberellic acid, are also included in this classification [96];
(c) Nitrogen-containing compounds, such as alkaloids (Alks), cyanogenic glucosides, and non-proteinogenic amino acids [97].

**Figure 3:**
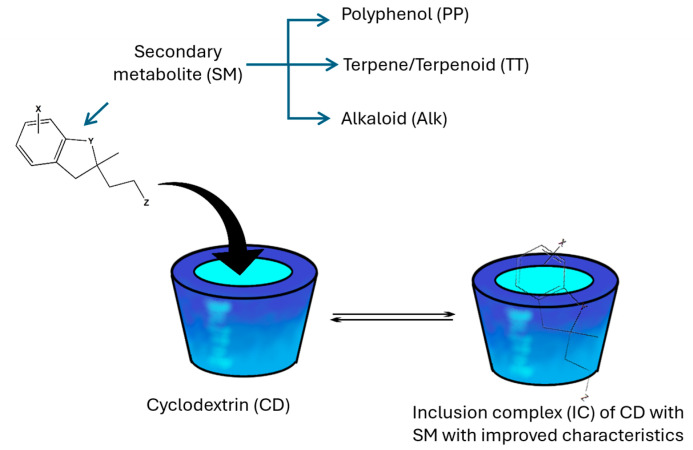
Schematic representation of IC formation between CD and SM (a virtual general structure is represented for SM). The three types of secondary metabolites that will be reviewed in this paper are polyphenols, terpenes and terpenoids, and alkaloids. The obtained inclusion complexes offer various advantages compared to the free secondary metabolites, enabling the exploration of various pharmaceutical applications, as detailed in this paper.

Figure 1 shows the recent increase in interest in publications on research topics related to cyclodextrins and secondary metabolites. CDs form inclusion complexes (ICs) with hydrophobic compounds, as illustrated in Figure 3 for secondary metabolites. Cs possess improved characteristics compared to the encapsulated compounds. Encapsulation allows for the modulation of the bioactive properties of SM and enhances their benefits, expanding the range of pharmaceutical applications.

## 4. Inclusion Complexes of Cyclodextrins with Secondary Metabolites

### 4.1. ICs of CDs-PPs with Pharmaceutical Applications

#### 4.1.1. Molecular Structure and Biological Activity of Polyphenols

**Scheme 1:**
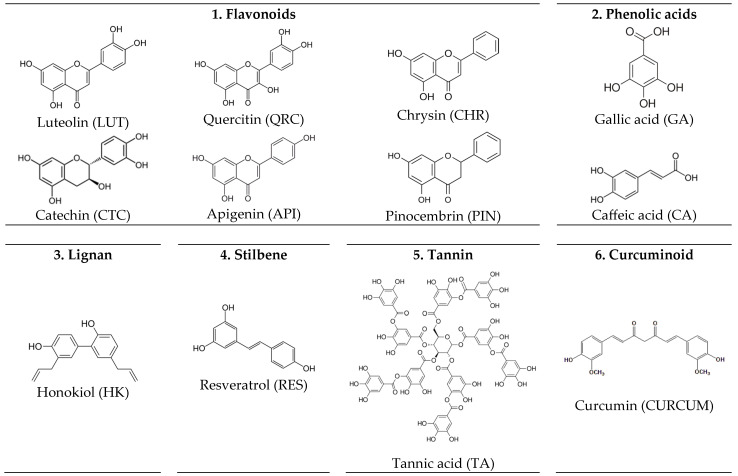
The molecular structures and classification of some polyphenols discussed in this review: flavonoids, phenolic acids, lignans, stilbenes, tannins, and curcuminoids.

Polyphenols (PPs), the most common phytochemicals, are biologically active compounds beneficial to human health. Chemically, they can be classified into five broad categories: phenolic acids, flavonoids, lignans, stilbenes, and tannins, all of which induce antibacterial activity as well as antioxidant and anti-inflammatory effects [98]. Selected examples of PP bioactivities include (a) quercetin, a known antioxidant [99]; (b) apigenin, known for its positive effects in diabetes, amnesia and Alzheimer’s disease, depression and insomnia, and cancer [100]; and (c) curcumin, which exhibits antioxidant, anti-inflammatory, and immune-regulatory activities, as well as protective or preventive effects on cancers, diabetes, and the liver, nervous, and cardiovascular systems [101]. The molecular structures of some polyphenols are shown in Scheme 1.

#### 4.1.2. CD Interactions with Polyphenols from Plant-Based Sources

Even though PPs possess important bioactive properties, their use is limited due to poor water solubility. The poor water solubility of polyphenols may be overcome by encapsulating them in CDs. Polyphenols are extracted from plants using selective, non-toxic solvents, sometimes mixed with certain cosolvents to achieve a more efficient process. Thus, cyclodextrins such as β-CD [102,103,104,105] and HP-β-CD, particularly when used in combination with ethyl alcohol, have been employed as green cosolvents [106]. CDs enable the simultaneous extraction and encapsulation of PPs, and some examples are provided in Table 1.

Recent studies report the preparation and characterization of inclusion complexes through the encapsulation of PPs from plant-based sources in cyclodextrins such as (i) β-CD [84,107,108,109,110,111,112,113] or its derivative, sulfobutyl ether-β-CD (SBE-β-CD) [114], either alone or in mixtures with other compounds based on maltodextrin, such as maltodextrin (MD), pectin, and sodium carboxymethylcellulose (CMC) (MD-CMC-β-CD CMC) [82], or MD-β-CD and gallic acid/gum Arabic (GA-β-CD) [115], and β-CD/whey protein isolate (WPI), WPI-β-CD [116]; (ii) HP-β-CD [65,83,117,118,119] and HP-β-CD in the form of nanofibers [120] or in the presence of liposomes [121]; and (iii) methyl-β-CD (M-β-CD) in the presence of a quaternary ammonium chitosan derivative (QA-CH), forming a composite compound of the QA-CH/M- β-CD type [85]. Also, α-CD, β-CD, and γ-CD were used as hosts for polyphenolic components from *Actinidia* leaves and red clover [86,122]. In the presence of CDs, the extraction of PPs was stimulated, having a positive impact on their antioxidant and antimicrobial activities. The main findings reported are presented in Table 2.

#### 4.1.3. Brief Review of Studies on ICs of CDs-PPs

The encapsulation process of polyphenols depends on the size of the cyclodextrin cavity and the molecular structure of the polyphenols. As shown in Table 3, different techniques, such as coprecipitation, freeze drying, and kneading, were used for the synthesis of ICs, and their confirmation was achieved using various methods, including SEM, FTIR, XRD, TG/DSC, DLS, UV-Vis spectrophotometry, and NMR.

Analyzing the information from Table 3, it is observed that most studies focus on the synthesis and analysis of inclusion complexes formed between β-CD and various polyphenols [63,70,73,74,76,80,81,123]. These are followed by studies involving the synthesis through interactions between HP-β-CD and various PPs [69,124,125]. Cyclodextrins and their derivatives offer good adsorption capacity for polyphenols under optimal encapsulation conditions. Adsorption (encapsulation) can be achieved through intermolecular bonds, especially non-covalent bonds such as hydrogen bonds and van der Waals forces. The desorption (release) process of polyphenols depends on their molecular structures, stereochemistry, and exposure factors, such as environmental composition and temperature.

The main findings of the studies focusing on CDs-PPs (pure or from plant-based extracts) indicate that the benefits of complexation are (a) improved solubility and bioavailability [74,111]; (b) improved stability (photo- and thermostability) and protection against degradation, allowing for prolonged shelf life of encapsulated natural compounds [69,73,76,83,114,116,125]; (c) masking of unpleasant taste [85]; (d) enhanced biological activities, such as antioxidant [65,70,73,77,82,84,86,107,110,111,112,114,115,116,122,124], antimicrobial [109], antidiabetic [84], and anticancer properties [63]; (e) controlled release behavior and targeted intestinal delivery, with pH resistance in the gastric environment [76,80]; and (f) the production of nutraceuticals (synthesis of natural products with increased antioxidant properties, in formulations with high stability, bioavailability and controlled release of antioxidants) [76,80,81,111,112,114,115,116,122,123] and cosmeceuticals (e.g., synthesis and delivery of natural anti-aging and antioxidant compounds with increased solubility and stability for human skin; use as natural UV filters) [113,118,119,121,124].

#### 4.1.4. Inclusion Complexes of PPs with CD-Containing Composites

To optimize the encapsulation and protection of PPs and to improve the properties and activity of PPs, various composite materials based on cyclodextrins were used. Inclusion complexes of quercetin (QRC), apigenin (API), and curcumin (CURCUM) with γ-CD incorporated into a metal–organic framework (MOF) led to improved solubility by 222/322/101 times and improved bioavailability by 18/19/145 times compared to those of free QRC/API/CURCUM [78].

Nanofibers containing HP-β-CD were used to incorporate a red vine leaf extract. Resveratrol exhibited increased solubility and better buccal penetration when incorporated into the nanofibers [120]. Vine leaf PPs and propolis PPs were complexed in HP-β-CD liposomes, creating a drug-in-cyclodextrin-in-liposome (DCL) system to deliver anti-aging compounds to human skin [121].

Epigallocatechin gallate (EGCG), in the presence of composite nanoparticles based on β-CD and corn starch (CS) (β-CD/CS) [126] and γ-CD/phospholipid-based nanovesicles (PBNs), γ-CD/PBN [127], led to the formation of inclusion complexes that improved photothermal stability and maintained the antioxidant activity (DPPH and ABTS assays) after 15 days. The pH-responsive behavior indicates that it would be a good candidate for oral targeted delivery systems [126]. A second study involving EGCG reports its encapsulation in γ-CD/phospholipid-based nanovesicles (PBN) [127]. The encapsulation of EGCG in nanovesicles and its controlled release were improved in the presence of γ-CD. Also, the complexation allowed for the masking of the bitter taste of EGCG [127].

The inclusion complex of ellagic acid (EA) and HP-β-CD incorporated in a CP-*g*-AMPS hydrogel was designed for orally controlled drug delivery, showing significant antioxidant activity (DPPH and ABTS) and excellent antibacterial properties against Gram-positive and Gram-negative bacteria [75]. The active compounds from pomegranate peel in the presence of HP-β-CD/pectin led to the formation of an inclusion complex that exhibited strong antioxidant activity, hypoglycemic potential, and significant antimicrobial activity against Gram-positive bacteria [128].

Other CD-containing composites that encapsulate PPs, for which in vitro or in vivo tests have been performed, are summarized in the section below (Section 4.1.5).

#### 4.1.5. In Vitro and In Vivo Studies

Results of in vivo and in vitro studies conducted on ICs of polyphenols or polyphenol-rich plant extracts and CDs are summarized in Table 2 and Table 3. Overall, the results indicated good antioxidant, antimicrobial, and anticancer activities, as well as enhanced bioavailability and bioactivity of PPs in encapsulated form.

Additional in vitro and in vivo studies have been performed using a wide range of polyphenols, such as tannic acid (TA) [129], curcumin (CURCUM) [130,131,132], caffeic acid (CA), gallic acid (GA), epicatechin (EC), quercetin (QRC) [131], epigallocatechin gallate (EGCG) [133,134], honokiol (HK) [79], and pinocembrin (PIN) [135], encapsulated in CD-containing composites.

Tannic acid, treated with a composite based on dexamethasone sodium phosphate (DSP) and poly-β-CD/DSP, formed an inclusion complex that exhibits a strong reactive oxygen species (ROS)-scavenging ability, capable of targeting the positively charged inflamed colonic mucosa in an inflamed mouse model, making it a potential therapeutic agent for the treatment of inflammatory bowel disease [129].

The self-assembled nanogels (NGs) based on lactoferrin (Lf) and carboxymethyl cellulose (CMC) were prepared for dual drug delivery of (a) pemetrexed (PMT), an antimetabolite with antitumor properties; and (b) of honokiol (HK), a herbal polyphenol. The entrapment efficiency of IC of HK and HP-β-CD into Lf-CMC NGs was 66.67%. PMT/HK-loaded Lf-CMC NGs demonstrated a controlled release profile for both drugs and indicated that the prepared nanogels might be promising vehicles for targeted combinatorial breast cancer therapy, as indicated in a mouse model [79].

Cyclodextrin-curcumin formulation added to the preservation solution in a DCD (donation after circulatory death) pig liver model did not improve ischemia–reperfusion injury severity, liver function, or survival [130]. The system, consisting of dual-network hydrogels (CS-GA/A-β-CD) with a snap structure, using crosslinked gallic acid-grafted chitosan (CS-GA) and aldehyde-β-CD (A-β-CD) including curcumin [131], was subjected to in vitro studies performed on cultures of microorganisms such as *S. aureus*, *E. coli*, and *P. aeruginosa*, as well as in vivo studies using a mouse full-thickness skin wound infection model. The results showed that (i) in vitro, the system exhibited excellent antimicrobial properties against *S. aureus*, *E. coli*, and *P. aeruginosa*; and (ii) in vivo, it promoted healing of acute bacterial infection wounds within 20 days [131].

The inclusion complexes of caffeic acid (CA), gallic acid (GA), epicatechin (EC), quercetin (QRC), and curcumin (CURC) incorporated in β-CD/PEG-600 hydrogels were studied in vitro on fibroblast cells and in vivo using a rat model. Due to the porous structure of CD-based hydrogels, they have demonstrated good injection capacity, drug-release ability, and antioxidant activity. Additionally, the hydrogels did not induce cytotoxicity in L929 fibroblast cells. The results suggested that the double drug-loaded β-CD-PEG-600-Ec hydrogel could potentially be used to prevent Asherman’s syndrome [132].

The nano-supramolecular delivery system, composed of β-CD supramolecular polymer (PCD) and thiolate chitosan (TCS) complexed with epigallocatechin gallate (EGCG), demonstrated potential as an antiallergic medicine for the long-lasting treatment of allergic rhinitis both in vitro on mast cells and in vivo in a rat model [133].

The epigallocatechin gallate (EGCG)/β-, γ-CD systems were applied to the influenza virus A/Bangkok/93/03, influenza A/PR8/8/34, influenza A/Aichi/2/68, and influenza B/Singapore propagated using MDCK cells. The complexes between CDs and EGCG showed effective inhibition against various viruses that require an adsorption step; therefore, this can be considered an effective tool for preventing viral infection [134].

The β-CD/nanoporous silicon composite complexed with caffeic acid and pinocembrin [135] was investigated in vitro on human umbilical vein endothelial cells (HUVECs) and in vivo on fertilized eggs of White Leghorn hens (*Gallus gallus*) for the evaluation of angiogenic activity [135]. The tests revealed that CA has stronger biological activity than PIN, especially in encapsulated form. Additionally, the composite can be considered a controlled drug-release system in antioxidant and antiangiogenic therapies for diseases such as cardiovascular diseases [135]. The inclusion complex based on tannins extracted from *Periploca angustifolia* roots and β-CD macrocyclic oligosaccharides demonstrated antioxidant and antihyperlipidemic effects in vivo [107]. Additionally, the chickpea sprout extract/β-cyclodextrin system was tested on a laboratory rat model. Pharmacokinetic analysis revealed that encapsulation increased the bioavailability of some isoflavones (formononetin and biochanin A), especially in the rat liver, as well as in other tissues and plasma [108].

#### 4.1.6. Theoretical Studies

The molecular docking calculations were applied to study the interactions between epigallocatechin-3-gallate (EGCG) and β-CD [64], indicating that entrapment takes place through stable π–π stacking interactions. The three aromatic rings of EGCG are entrapped in β-CD, and eight hydrogen bonds help the encapsulation [64].

Additionally, the molecular docking method was used to demonstrate the host–guest interactions between certain cyclodextrins and their derivatives, namely α-CD, β-CD, γ-CD, HP-β-CD, dimethyl-β-CD (DM-β-CD) and glucosyl-β-CD (GL-β-CD) and polyphenols such as catechin, iso-liquiritigenin, luteolin, puerarin, rutin, and curcumin [71].

The interactions between the tea polyphenol epigallocatechin gallate (EGCG) and various cyclodextrins such as α-, β-, γ-CD, and the γ-derivative (HP-γ-CD) were investigated using umbrella sampling techniques. The theoretical calculations revealed that HP-γ-CD had the highest affinity for EGCG [136].

In 2023, Aree [123] studied the inclusion complexes of three predominant polyphenols in apples, namely phloretin (PRT), phlorizin (PRZ), and ferulic acid (FEA), with β-CD and α-CD. This study focused on three complexes, namely β-CD-PRT (1), β-CD-PRZ (2), and α-CD-FEA (3). The encapsulation of PPs in CDs led to increased water solubility and stability of polyphenols. The stability of the complexes varied as follows: β-CD-PRZ > β-CD-PRT > α-CD-FEA. According to density functional theory (DFT), the number of intermolecular bonds that can form between PPs and CDs followed the same trend: β-CD-PRZ (seven intermolecular bonds), β-CD-PRT (three intermolecular bonds), and α-CD-FEA (one intermolecular bond) [123].

### 4.2. ICs of CDs-TTs with Pharmaceutical Applications

#### 4.2.1. Molecular Structure and Biological Activity of Terpenes and Terpenoids

Terpenes are SMs obtained through the polymerization of isoprene units, while terpenoids are their derivatives, containing various functional groups, typically including oxygen atoms. Terpenes can be classified as hemiterpenes (C_5_), monoterpenes (C_10_), sesquiterpenes (C_15_), diterpenes (C_20_), triterpenes (C_30_), and tetraterpenes (C_40_). Gathering around 80,000 compounds, TTs are the largest class of natural compounds, accounting for approximately 60% of plant SMs [137,138].

**Scheme 2:**
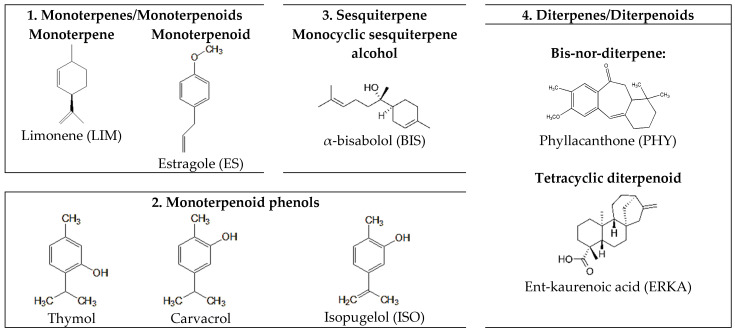
The molecular structures and classification of some terpenes and terpenoids discussed in this review.

The molecular structures of some terpenes and terpenoids discussed in this review are shown in Scheme 2.

The main components of essential oils have a positive impact on human health. Numerous in vivo and in vitro studies report their diverse beneficial biological activities, including antidiabetic, anticancer, antimicrobial, antioxidant, anti-inflammatory, antiallergic, neuroprotective, anticoagulation, sedative, and analgesic effects [94].

#### 4.2.2. CDs Interactions with Pure TTs and TTs Rich Extracts from Plant-Based Sources

Pure TTs or TT-rich extracts have been used as guests for CDs, with the most intensively studied being ICs of β-CD [66,139,140,141,142,143]; however, ICs of HP-β-CD [144,145] and SBE-β-CD [141] have also been reported. Encapsulation efficiency depends on the method of synthesis [66,139], and studies show that it increases when certain emulsifiers, such as ethanol or glycerol, are added during the encapsulation process [140]. Some examples are provided in Table 4.

#### 4.2.3. Inclusion Complexes of TTs with CD-Based Composites

Composites containing CDs have been used for the encapsulation of PHY in magnetic nanoparticles [146] and for the encapsulation of a TT-rich extract in nanosponges [147]. The results of in vivo studies conducted using complexes of TTs with CD-based composites are presented in the next section, Section 4.2.4.

#### 4.2.4. In Vitro and In Vivo Studies

In vivo and in vitro studies conducted on ICs of TTs or TT-rich plant extracts and CDs are summarized in Table 4. Overall, the results indicated good anticancer activity for the complexes [139,141]. Other observed effects indicated the potential of ES to be used in the therapy of multidrug-resistant bacteria as an adjuvant to other antibiotics [66].

In vivo studies indicate that encapsulation allows for a reduction in the therapeutic dose of TTs. In a complete Freund’s adjuvant-induced arthritis mouse model, bisabolol (BIS) has antiarthritic, anti-inflammatory, antioxidant, and antihyperalgesic effects. BIS complexation with β-CD improved its pharmacological efficacy. Therefore, the therapeutic dose may be reduced, and the associated toxicity will diminish [142]. A similar effect was observed when isopulegol (ISO) was complexed with β-CD. Complexation increased the ISO antinociceptive effects and bioavailability in mice, allowing its use at a lower therapeutic dose [143]. Limonene (LIM) demonstrated anti-nociceptive and anti-inflammatory effects in vivo when tested on rats and mice, making it a promising molecule for the treatment of oro-facial pain. LIM complexation with HP-β-CD allowed for a decrease in the therapeutic dose of LIM [144].

In vitro studies have also been performed on CD-containing composites. PHY has been loaded into magnetic iron oxide (Fe_3_O_4_) nanoparticles containing β-CD with good loading efficiency. The obtained nanoparticles showed a slow and sustained release of PHY, demonstrated a stronger anticancer effect compared to free PHY, and are suitable for theranostic applications in photoacoustic microscopy [146]. An ethyl acetate-rich extract high in TTs from *Boswellia carterri* was loaded into nanosponges containing HP-β-CD and epichlorohydrin-β-cyclodextrin (EPI-β-CD), achieving very good encapsulation efficacy (over 98%). The nanosponge obtained using HP-β-CD showed promising therapeutic effects on upper and lower respiratory ailments in female Wistar albino rats with respiratory distress [147].

#### 4.2.5. Theoretical Studies

Theoretical studies investigate the interaction of β-CD with certain TTs [66,139,141,148] and of SBE-β-CD with PHY [141], using molecular docking [66,139,141], molecular predictive optimization [66], and the GFN2-xTB multi-equilibrium approach [148]. Calculations indicate that β-CD interacts with high affinity with estragole (affinity energy: −5.1 kcal/mol) [66] and ERKA (−6.4 kcal/mol) [139]. Molecular docking calculations, corroborated by NMR and FTIR analysis, indicate that the complexation of PHY by β-CD and SBE-β-CD probably takes place with the participation of H-3, H-5, and H-6 from the CDs with PHY [141]. Other studies report that the calculated distance from ERKA to β-CD was 2.04 Å [139] and that the guest orientation of thymol is opposite to that of carvacrol [148].

### 4.3. ICs of CDs-Alks with Pharmaceutical Applications

#### 4.3.1. The Molecular Structure and Biological Activity of Alkaloids

**Scheme 3:**
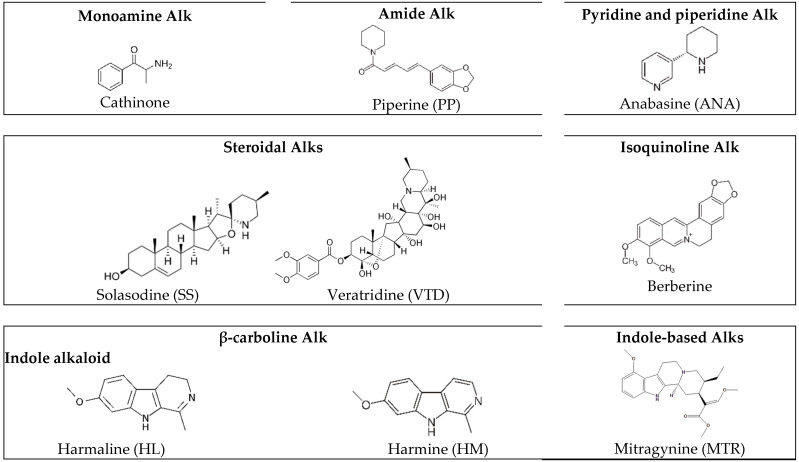
The molecular structures and classification of some alkaloids discussed in this review.

Alkaloids are a class of secondary metabolites that contain nitrogen atoms, typically within a heterocycle. They have diverse chemical structures and are classified into different groups based on the structure of the heterocycles they contain. Alkaloids have alkaline properties and may modify the activity of the central nervous system (CNS). They have antiviral, antibacterial, anti-inflammatory, analgesic, insecticidal, and anticancer properties [149,150], but they also possess high toxicity. The toxic effects of alkaloids range from mild (e.g., nausea and vomiting) to severe (e.g., psychosis, paralysis, arrhythmias, and sudden death) [151]. In cases of chronic consumption, some alkaloids may have teratogenic and carcinogenic effects [151]. Overconsumption of arecoline is carcinogenic and may lead to tumor formation [152]. The molecular structures of some alkaloids discussed in this review are shown in Scheme 3.

#### 4.3.2. CDs Interactions with Alks and Alk-Rich Extracts from Plant-Based Sources

Various alkaloids have been used as guests for CDs. Unlike previously presented studies, complexation of alkaloids using β-CD [153,154,155,156,157] has been less intensively studied than complexation using its derivatives: HP-β-CD [158], SBE-β-CD [159,160,161], methylated CDs [154,155], and other derivatives [162,163,164,165]. Examples are provided in Table 5.

From Table 5, the main conclusions regarding CD-Alk complexes are as follows:

– Main properties of CD-Alk complexes indicate increased water solubility [156], stability, and bioavailability of CD-Alk complexes compared to free alkaloids [153]. Possible applications may be found in the case of mitragynine, an alkaloid with analgesic properties [156]. Lys-β-CD proved to be an effective complexation agent for berberine, yielding a potential sustained-release system, with applications in drug delivery and biomedical fields [160]. Other studies investigate the association strengths between SBE_6_._4_-β-CD (SBE-β-CD with an average of 6.4 degrees of substitution) and various Alks [161] or between certain Alks (piperine, veratridine) and β-CD or its derivatives [155,156,157]. These studies indicated that SBE-β-CD forms more stable complexes with alkaloids than β-CD. A higher complex stability was also obtained when medium cavity-sized, negatively charged CDs were used for complexation [165,166].
– Fluorescence spectroscopy allows for the analysis of fluorescent alkaloids. For example, harmaline (HL) and harmine (HM) exhibit superposed fluorescence spectra. Synchronous fluorescence measurements, following the inclusion of HL and HM in HP-β-CD, allowed for the separation of overlapping signals and the simultaneous determination of these Alks in various matrices, with high sensitivity and precision [158]. Other studies present the results of fluorescence spectroscopy measurements of berberine (BBR) and SBE_10_-β-CD complex [159,160]. Complexation with SBE_10_-β-CD increases the fluorescence intensity of BBR (~190-fold). In the presence of Cd^2+^ ions, the supramolecular complex SBE_10_-β-CD-BBR enables the detection of adenosine triphosphate (ATP), suggesting its potential use as a biosensor for ATP [159]. Other studies have investigated the potential of the same complex, SBE_10_-β-CD-BBR, to serve as a biosensor for the cancer biomarker spermine. Spermine is a metabolite whose concentration increases in urine and serum in the presence of malignant cells in the body [160].
– The strong complexation ability of CDs for toxic alkaloids indicates that CDs may have potential applications as antidotes in cases of alkaloid intoxication, including veratridine [157], solasodine [163], kratom alkaloids [164], and cathionine and its derivatives [165].

#### 4.3.3. Inclusion Complexes of Alks with CD-Containing Composites

A magnetic silica composite functionalized with β-CD was prepared and used to determine the Alk content in poppy seed infusions rich in opium alkaloids, using magnetic solid-phase extraction [166]. Other CD-containing composites complexed with Alks, studied in in vivo and in vitro models, are described in the next section, Section 4.3.4.

#### 4.3.4. In Vitro and In Vivo Studies

HP-β-CD/polyacrylamide (PAA) hydrogels were prepared and loaded with carbazole (Carb) through the co-incubation of Carb and the hydrogels in ethanol. The loaded hydrogels showed antibacterial properties, both in vitro and in vivo, and accelerated the healing process of bacteria-infected wounds in mice, making them good candidates for wound healing applications [167]. Another composite with applications in the treatment of infections was obtained using the *Peganum harmala* L. harmala alkaloid-rich fraction (HARF), which was complexed with HP-β-CD and then co-encapsulated with ascorbic acid into nanoparticles of poly(lactic-co-glycolic acid) (PLGA) coated with polyethylene glycol. Complexation enhanced the antibacterial and antiviral activities of HARF and yielded a biocompatible composite [168].

#### 4.3.5. Theoretical Studies

Theoretical studies have primarily investigated the interactions of native CDs: α-CD, β-CD, and γ-CD [153,157], and their derivatives, such as HP-β-CD [154], SBE-β-CD [157], Lys-β-CD [162], and sugammadex [163], mainly using molecular docking calculations. These theoretical results were in good agreement with experimental data, confirming that SBE-β-CD and γ-CD bind more strongly to VTD than β-CD [157]. However, when complexed with anabasine, β-CD is better suited for complexation than α-CD and γ-CD [153]. Another study showed that piperine interacts more strongly with randomly methylated-β-CD than with HP-β-CD [154]. Kalydi et al. indicated that the complexation of solasodine (SS) by sugammadex takes place through intermolecular ionic interactions between the secondary amino group of SS and the carboxylate ends of the sugammadex side chains [163].

## 5. Pharmacokinetics and Pharmacodynamics of Cyclodextrin Inclusion Complexes with SMs

The pharmacokinetics of inclusion complexes involve the simultaneous metabolism of the complexes, as well as of the cyclodextrins and secondary metabolites. Thus, the pharmacokinetic parameters of CDs-SMs depend on the type and number of host–guest interactions. Hydrogen bonds are stronger than van der Waals forces, ensuring greater stability of the inclusion complex and thus leading to limited metabolism of the latter and a relatively high possibility of being eliminated unchanged. The model of animals subjected to the study, such as mice, rats, or dogs, constitutes a set of factors and characteristics that can influence the pharmacokinetic parameters.

Also, the pharmaceutical parameters of the host-cyclodextrins, such as molecular mass, approximate cavity diameter (Å), and molar degree of substitution, are involved in the evaluation of both the pharmacokinetic parameters of cyclodextrins and those of inclusion complexes [169]. In the case of oral or topical administration of β-cyclodextrin in rats, the half-life (t_1/2_) ranged from 23.9 min to 50.2 min, and the clearance (CL) reached values between 204 mL/h kg and 372 mL/h kg. For HP-β-CD administered orally, t_1/2_ was similar at 24 min, and CL increased significantly to 512 mL/h kg [169], meaning that at the same CD concentration, the plasma clearance rate (mg/min) is higher for HP-β-CD than for β-CD.

Myricetin, a flavonoid with a wide extraction potential from tea, berries, fruits, and medicinal herbs, complexed with HP-β-CD, formed an inclusion complex with an oral bioavailability in rats that was 9.4 times higher than that of free myricetin [170]. However, in the physiological environment, the interactions are more complex, taking into account that plasma proteins simultaneously compete with CDs for drug binding. In the case of oral administration, an excess of cyclodextrins can hinder drug absorption from the gastrointestinal tract, potentially leading to a relatively high elimination of the unmetabolized drug [171]. Thus, in complicated interconnections, the pharmacokinetics will be determined by the binding of the drug to plasma proteins [171].

The rat plasma pharmacokinetics of isoflavones from chickpea sprout extracts (CSE), whether free or complexed with β-CD (CSE/β-CD) [108], showed that after oral administration, the plasma concentrations of formononetin (FMN) and biochanin A (BCA) reached a maximum value (Cmax) at 0.25 h [108]. The half-lives (t_1/2_-h) and Cmax (ng/mL) increased for the CSE β-CD systems, while CL (L/h) decreased compared to the isoflavones from CSE not bound to cyclodextrin. Additionally, the BCA inclusion complex had a longer half-life (7.33 h) and a higher Cmax of 22.45 ng/L compared to the FMN inclusion complex, which had a half-life of 4.51 h and a Cmax of 14.37 ng/L, respectively. Therefore, β-CD can enhance the low solubility of the two isoflavones, improving their bioavailability in rats [108].

The pharmacodynamics demonstrated that the complexation of ursolic acid (a terpenoid) with HP-β-CD resulted in increased antiproliferative activity against melanoma cell lines compared to unbound ursolic acid. Also, the HP-β-CD/curcumin–liposome inclusion complex inhibited the proliferation of breast cancer cells in animal models [172]. Inclusion complexes of saikosaponin-d and betulinic acid with HP-β-CD and β-CD improved the anticancer activity of the two terpenes. On the other hand, encapsulation of betulinic acid in β-CD diminished the proliferation of human breast cancer cells. Also, the anticancer activities of the alkaloids from *Camptotheca acuminata*, namely camptothecin and luotonin A, were improved by their microencapsulation in β-CD and HP-β-CD [172].

The curcumin-HP-β-CD inclusion complex has antiepileptic effects and can contribute to the control of seizure frequency and intensity [173,174]. Curcumin formulation with HP-β-CD led to a 2.8-fold increase in bioavailability [173]. The antiepileptic effect was more pronounced in pentylenetetrazole-induced zebrafish and mouse models [173,175].

Polyphenols such as quercetin, curcumin, rosmarinic acid, ferulic acid, and resveratrol have neuroprotective properties, inducing antioxidant, anti-inflammatory, and anti-apoptotic effects. They stimulate cognitive function and memory, and generate a positive impact on lability, hallucinations, depression, and dementia [176]. Alkaloids such as huperzine A and caffeine may help manage Alzheimer’s disease by enhancing memory and attention [176,177]. Additionally, significant results were obtained showing that ginkgolides (terpene lactones) can relieve the symptoms of depression and dementia [176]. Depressive disorder is a common condition that affects children, adults, and the elderly. To address the slow therapeutic response to antidepressant drugs, cyclodextrins can serve as an alternative to circumvent these limitations and enhance the physicochemical and pharmacological properties of these drugs.

Sometimes, inclusion complexes of cyclodextrins with secondary metabolites can induce higher toxicity than the unformulated constituents. Thus, caffeine formulated with α-, β-, and γ-cyclodextrins induced greater toxicity in zebrafish embryos compared to free, uncomplexed caffeine and the CDs. It was found that the cytotoxicity was not caused by the increase in caffeine concentration in the zebrafish embryos, as similar levels were observed regardless of its form of administration (uncomplexed or complexed with CDs). The increased toxicity of the caffeine-CD complex could be caused by a synergistic interaction between caffeine and cyclodextrins, resulting in the potentiation of the effects of CDs on caffeine [178].

## 6. Risks and Limitations

The interactions between CDs and bioactive compounds from plants, especially fruits, can be affected by various factors, including their storage conditions and duration. Improper storage conditions, such as high temperatures and excessive light, can favor the decomposition of secondary metabolites over time, potentially leading to unreproducible and unsatisfactory results. Since plant extracts are multicomponent systems, there is a risk of forming additional inclusion complexes alongside the targeted ones. In this regard, HPLC constitutes an advanced analysis technique that should be performed before and after complexation. It provides information on the stoichiometry and association constants of the CD-SM complexes.

To obtain stable inclusion complexes, cyclodextrins and secondary metabolites must exhibit compatibility to participate in host–guest interactions; thus, their optimal selection constitutes the first stage of the research. The possibility of forming CD-SM bonds must be studied, depending on the molecular structure of the metabolite. SMs should provide functional groups capable of forming intermolecular bonds (e.g., hydrogen bonds) with the -OH internal groups of the cyclodextrin macrocycle or heteroatoms possessing vacant orbitals that could be occupied by the lone electron pairs belonging to the oxygen atoms linking the α-D-glucopyranoside structural units.

Polyphenols have hydroxyl groups that can engage in intermolecular bonds with cyclodextrins; alkaloids can act as guests through nitrogen atoms; and the steric configuration of terpenes and terpenoids may affect their ability to form complexes with CDs.

Thus, it was reported that the interaction of caffeine with HP-CDs and M-CDs in water at 298.15 K, studied by calorimetry, spectroscopy, and solubility methods, did not lead to the formation of stable complexes. Changing the size of the cyclodextrin cavity did not favor the formation of more stable intermolecular bonds and implicitly did not enhance the ability of cyclodextrin to form the caffeine-CD complex [179]. The inclusion complexes between β-CD and caffeine, along with the biophenols present in olive oil (tyrosol, hydroxytyrosol, homovanillic acid, 3,4-dihydroxyphenylacetic acid, and protocatechuic acid), were investigated using NMR. It was found that the incorporation of biophenol molecules into the β-CD cavity prevailed over caffeine encapsulation, with binding constants that were 10 to 40 times higher than those of caffeine, in a molar ratio of 1:1, in an aqueous solution. Consequently, inclusion complexes were preferentially formed with biophenols, to the detriment of those with caffeine [180]. Therefore, the preparation method can pose a risk for the formation of inclusion complexes. Operating parameters, such as mixing and/or stirring, time, and temperature, are essential factors in the synthesis of CD-SM complexes. The methods applied for the preparation of ICs can have many advantages, but some optimizations are also welcome, such as the addition of appropriate additives to increase complexation efficiency.

Theoretical studies are of great relevance, but for consistent results, they must be supported by experimental data. Theoretical studies can accurately designate the type and number of bonds formed between CDs and SMs, thereby confirming the formation of inclusion complexes, as well as their binding to proteins, enzymes, and lipids, but they cannot provide assessments of their activity.

Complexation additives (CADs) can enhance the formation of inclusion compounds and, consequently, improve complexation efficiency. However, there is a risk that they may compete with the SM for binding to cyclodextrin, which could create uncertainty regarding the formation and structure of the inclusion complex. The CAD/CD mixture should be designed as a coherently organized composite that enables the preferential encapsulation of the secondary metabolite within the cyclodextrin cavity. Additionally, complexation additives must be non-toxic to avoid creating additional problems, especially in in vivo tests. CAD should involve biologically active compounds that are engaged in the absorption or metabolism of drugs.

In vivo studies target several animal species that tolerate a drug differently when it is administered orally or parenterally. Thus, the behavioral deviations of the model species observed after the first drug administration must be correlated with the mortality rate, as this information constitutes a primary indication for further research. Parenteral administration presents certain advantages, including precise dosing of the drug and the attainment of a rapid effect.

## 7. Perspectives

Inclusion complexes of cyclodextrins with secondary metabolites (CDs-SMs) still present many challenges. Therefore, the design and development of advanced, non-toxic systems that offer the advantages of solubility and bioavailability, as well as the extension of the controlled release time of the secondary metabolite, are encouraged [173]. There is a need to develop concepts that improve the clinical application of cyclodextrin–secondary metabolite-based systems. Approved commercial products use native CDs, such as α-CD and β-CD, as well as their derivatives, including 2-HP-β-CD, methylated beta-cyclodextrin (M-β-CD), and SBE-β-CD [170].

CDs and their derivatives can offer encouraging prospects for the release of insoluble secondary metabolites or SMs with other undesirable properties. The development of new CD-SM systems that can be marketed is supported, taking into account that only conventional formulations have been addressed to humans.

Moreover, CDs are promising as “smart substances”, which can lead certain guests to target cells that have been modified by diseases. CDs also have the ability to prolong the release time of an encapsulated secondary metabolite, leading to its controlled release and action for a longer time. On the other hand, CDs are biodegradable substances that can be broken down within the body, facilitating their almost complete elimination without significant accumulation in the viscera or tissues.

However, to avoid a negative impact on research performance, it is imperative to accurately establish the binding mechanism between CD-SM and to exclude other interactions between cyclodextrin and potential ingredients in the formulation [170].

In order to prevent irritation of the tissue from the buccal cavity, the synthesis of inclusion complexes that mask the astringent or bitter taste is encouraged.

## 8. Conclusions

A large number of studies have investigated the use of CDs as inclusion agents through host–guest interactions with secondary metabolites. Some properties and applications are common to the three classes of SMs described in this paper, while others are rather specific to each SM class. The general effects that can be attributed to the three classes of SMs consist of increased solubility, bioavailability, photostability, thermal stability, pharmacological efficacy, and biological activity; decreased therapeutic dose and toxicity; controlled drug release; and the synthesis of composites capable of controlled drug release. The specific effects of certain classes of SMs have been studied more intensively, reporting that PPs have antioxidant effects when used as nutraceuticals and as cosmeceutical agents. They also exhibit anti-aging properties and act as natural UV filters. TTs confer antinociceptive and anti-inflammatory effects, while Alks have analgesic properties. CDs have been studied as potential antidotes in cases of Alk intoxication, and CD-Alk complexes may be used as biosensors for metabolites or tumor markers.

CDs are also used as green cosolvents for the separation of secondary metabolites from complex natural extracts, acting as both extraction and encapsulation agents. Many studies have described CDs as components of composites that facilitate the inclusion of PPs, TTs, or Alks for various applications. Most of these systems allow for controlled drug release or enhance bioactivities of significant interest, such as anticancer properties or the ability to potentiate antimicrobial action.

Certain papers have included theoretical studies, such as molecular docking modeling, to determine metabolite–host interactions. Molecular docking also indicates the affinity energy involved in these interactions, providing information on the stability of micro-aggregates and allowing for a meaningful comparison of data between ICs of different CDs with the same metabolite. Such data help researchers identify the best inclusion agent before starting their experimental investigation.

## Figures and Tables

**Table 1:** CDs as green cosolvents for the extraction of PPs from plants.

Plant-Based Sources	Green Cosolvent	Extraction Method	Ref.
*Colocasia esculenta* L. leaves	β-CD	ultrasonication of leaves with an ethanolic solution of β-CD	Christou et al., 2024 [102]
post-distillation rosemary solid residues		solid–liquid extraction with an aqueous solution of β-CD	Tsitlakidou et al., 2024 [103]
avocado peel		maceration plus β-CD	Martínez-Gutiérrez 2023 [104]
pomelo peel (*Citrus maxima*)		microencapsulation by spray drying	To et al., 2022 [105]
*Gentiana asclepiadea* L.; willow gentian underground parts, an unexploited herbal tea by-product	HP-β-CD	ultrasound-assisted extraction	Jovanović et al., 2022 [106]

**Table 2:** The benefits of inclusion complexes of cyclodextrins with some polyphenols from vegetable sources.

Plant-Based Sources of PPs	CDs	Main Findings/Benefits of Complexation	Reference
Tannins from *Periploca angustifolia* roots	β-CD	Encapsulation efficacy of 70% in β-CD was obtained; tannin release with enhanced antioxidant and antihyperlipidemic activities of ICs; pure and encapsulated tannins demonstrated effective inhibition of pancreatic lipase activity.	Diwani et al., 2022 [107]
Chickpea sprouts isoflavones		The content of isoflavones in chickpeas increased through sprouting; efficient extraction of components with antioxidant activity in the presence of β-CD; increased bioavailability of extracted flavonoids through encapsulation.	He et al., 2023 [108]
Red propolis (Prop)		Antimicrobial activity against microorganisms from the oral cavity.	Moreira et al., 2022 [109]
Blackthorn fruits		Antioxidant capacity increased by over 60%; β-CD enables simultaneous extraction and encapsulation.	Blagojević et al., 2023 [84]
Fenugreek Seeds (*Trigonella foenum-graecum*)		β-CD-assisted extraction allowed for a higher yield of components with antioxidant, anti-inflammatory, and hypoglycemic effects.	Frangopoulos, 2022 [110]
Nettle leaves (*Urtica dioica* L.)		Extracts obtained using spray encapsulation in the presence of β-CD contained the highest phenolic content and antioxidant activity; encapsulation increased the bioavailability of polyphenols.	Cegledi et al., 2022 [111]
Green rooibos (*Aspalathus linearis*)		An increase in β-CD concentration generated an increase in the extraction yield of polyphenols, which correlated with an increase in antioxidant activity.	Vhangani et al., 2022 [112]
Onion peel (OP) extract rich in quercetin and resveratrol		Encapsulation efficiency: 91.8%; microencapsulation protected OP extract, prolonging its shelf life; OP extract, whether in its natural form or encapsulated, can be used as a natural sunscreen, allowing for the replacement of synthetic sunscreens.	Messias et al., 2023 [113]
Red Clover aerial parts (*Trifoliu pratense* L.)	β-CD; γ-CD	Significant increase in extracted total phenolic content (TPC): 20.29% increase in aqueous samples, using β-CD, and 22.26% increase in ethanolic samples, using γ-CD; a direct correlation was observed between TPC and antioxidant activity.	Kazlauskaite et al., 2022 [122]
Tea polyphenols (TP)	SBE-β-CD	Improved antioxidant activity, α-glucosidase scavenging ability, and thermal stability; designed as a nutraceutical with antioxidant and hypoglycemic properties.	Li et al., 2023 [114]
Aqueous extract of *Bauhinia ungulata* var. *obtusifolia* leaves	MD-CMC-β-CD -	Antioxidant and antidiabetic properties of identified phenolic compounds with thermal stability, such as p-coumaric acid, chlorogenic acid, rutin, and isoquercitrin.	Remígio et al., 2024 [82]
Laurel (*Laurus nobilis* L.) leaves	β-CD; MD-β-CD; GA-gum Arabic-β-CD	β-CD was less efficient than β-CD + MD/GA in preserving the flavonols during digestion; β-CD + MD induced optimal solubility, hygroscopicity, and antioxidant capacity.	Dobroslavić et al., 2023 [115]
Cashew nut testa (CNT), an underutilized cashew by-product rich in polyphenols	WPI-β-CD-	Higher solubility, stability, antioxidant activity, and increased controlled release of PPs in the encapsulated form.	Sruthi et al., 2024 [116]
Propolis	HP-β-CD	Complexation enables a good solubility of polyphenols; stable IC with good bio-accessibility of main PPs at the small intestine level.	Perak et al., 2023 [117]
*Cajanus cajan* (*C. cajan*) leaves		Inhibitory effects on Gram-positive bacteria and strong antioxidant activity; might be applied for pharmaceutical preparations due to their high load capacity, high solubility and increased biological activity.	Fu et al., 2023 [65]
*Fagopyrum esculentum* Moench (common buckwheat)		Positive impact on the extraction efficiency of rutin, quercetin, and total polyphenols and on their stability when exposed to stress conditions.	Radan et al., 2023 [83]
Two *Helichrysum italicum* extracts: OPT-1 (rich in phenolic acids) and OPT-2 (rich in total phenols and flavonoids)		In most of the assays, the antioxidant and cosmeceutical activities of tested compounds were better than those of positive controls.	Jakupović et al., 2023 [118]
*Satureja montana* L.	HP-β-CD	The extracts displayed good anti-elastase and excellent anti-hyaluronidase activity, making them suitable components of natural cosmetic products.	Marijan et al., 2023 [119]
Red vine leaf	nanofibers containing HP-β-CD	In the nanofiber combination, resveratrol had increased solubility and better buccal penetration.	Paczkowska-Walendowska et al., 2023 [120];
Vine leaves PPs and propolis PPs	HP-β-CD liposome	PPs/HP-β-CD/liposome system delivered anti-aging compounds for human skin.	Athanasopoulou et al., 2024 [121]
Bergamot essential oil (BEO) (rich in polyphenols and limonene)	M-β-CD/QA-Ch	A conjugate, BEO/QA-CH/M-CD was obtained, allowing for the stabilization of volatile compounds of BEO and the elimination of its unpleasant taste; conversion of the oil into a solid dosage form (powders, granules, tablets); better protection of PPs and limonene.	Zambito et al., 2022 [85]
*Actinidia* leaves (Geneva, Jumbo, Ken’s Red, Kijivska Hibridna, and Sentyabraskaya)	α-CD β-CD γ-CD	*Actinida* leaves main constituents: quercetin, rutin, epicatechin, chlorogenic acid, and kaempferol; the highest biological activity (antioxidant and enzyme inhibition assays) was found in Ken’s Red variety; extraction in the presence of CDs increases the biological activity of Ken’s Red leaves.	Sip et al., 2022 [86]

**Table 3:** Encapsulated polyphenols in host cyclodextrins, preparation, analysis methods, and main benefits of ICs.

Polyphenol	CD or CD-Derivative	Preparation Methods	Confirmation Techniques	Reference
Luteolin (LUT)	β-CD	Freeze-drying method	FTIR, XRD, DSC, UV-Vis	Song et al., 2024 [70]
Catechin (CAT)		Kneading Freeze-drying method	SEM, ESI-MS/MS	Ntuli et al., 2022 [73]
Epigallocatechin-3-gallate (EGCG)				
Gallic acid (GA)				
Alkyl gallates (dodecyl gallate, butyl gallate, octyl gallate, and ethyl gallate)		Freeze-drying method	HPLC-UV analysis	Cai et al., 2024 [80]
Alkyl gallates (butyl, propyl, ethyl, and methyl gallates)		Freeze-drying method	NMR, SEM, XRD, FTIR	Cai et al., 2024 [81]
Phloretin (PRT) Phlorizin (PRZ) Ferulic acid (FEA)		Solvent evaporation of concentrated solutions containing β-CD and PPs	XRD	Aree 2023 [123]
Curcumin (CURCUM)		Coprecipitation	SEM, XRD, FTIR	Song et al., 2022 [74]
Quercetin (QRC)		Nanoprecipitation, lyophilization	FTIR, NMR, SAED *, XRD	Rajamohan et al., 2023 [63]
Hydroxytyrosol (HT)	α-CD, β-CD	Freeze-drying method	UV-Vis, NMR, DSC, FTIR	Stergiou et al., 2022 [76]
Phloroglucinol (PGL)	γ-CD	Co-dissolution and freeze drying	FTIR, TG, DTA, XRD	Catarino et al., 2022 [77]
Chrysin (CHR)	HP-β-CD	Kneading and coprecipitation	FTIR, TG, SEM, NMR	Lavania et al., 2023 [69]
Pterostilbene (PTS)	HP-β-CD	Freeze-drying method	XRD, SEM, NMR, TG, DSC	Yang et al., 2024 [124]
Resveratrol (RES)	HP-β-CD	Spray-drying technique	HPLC, FTIR, DSC, XRD	Yang et al., 2022 [125]

* SAED: selected area (electron) diffraction.

**Table 4:** The benefits of ICs of CDs and pure TTs or TT-rich extracts, with pharmaceutical applications.

TTs	CDs	Main Findings/Benefits of Complexation	Reference
Estragole (ES) (Terpenoid)	β-CD	Encapsulation of ES in β-CD was calculated to be 25.45%. In vitro: β-CD-ES potentiates the antibiotic effect of some compounds but does not exhibit antibiotic activity when administered alone.	Costa et al., 2024 [66]
*Mikania glomerata* leaves extract rich in Ent-kaurenoic acid (ERKA) (Terpenoid)		ERKA inclusion in β-CD using the malaxation method (65.37%) was superior to inclusion using the co-evaporation method (13.64%). In vivo: antitumor activity in mice and low systemic toxicity.	Alves et al., 2023 [139]
*Lavandula stoechas* essential oil (terpenes and terpenoids)		Encapsulation efficiency increased when ethanol and glycerol were used as green emulsifiers during the encapsulation process. In vivo: encapsulation increased the thermal stability of *L. stoechas* essential oil and its insecticidal effect on adult mosquitoes.	Ez-zoubi et al., 2022 [140]
Phyllacanthone (PHY) (terpene) isolated from stem barks of *Cnidoscolus quercifolius*	β-CD SBE-β-CD	ICs of PHY with β-CD and SBE-β-CD have been obtained; complexation improves the water solubility of PHY. In vitro studies: free and complexed FHY mitigate the growth of melanoma cells.	Alves et al., 2023 [141]

**Table 5:** The benefits of ICs of CDs and pure Alks or Alks-rich extracts, with pharmaceutical applications.

Alks	CDs	Main Findings/Benefits of Complexation	Reference
Anabasine (ANA)	β-CD	Increased stability and bioavailability of ANA.	Muldakhmetov et al., 2022 [153]
Harmaline (HL) and harmine (HM)	HP-β-CD	Fluorescence spectroscopy indicates that complexation allows the simultaneous determination of HL and HM from various matrices.	Abdelazim et al., 2023 [158]
Berberine (BER)	SBE_10_-β-CD	Fluorescence spectroscopy indicated that SBE_10_-β-CD-BBR-Cd^2+^ may serve as a biosensor for the bio-analyte ATP.	Chakraborty et al., 2022 [159]
	SBE_10_-β-CD	Fluorescence spectroscopy indicated that SBE10-β-CD-BBR may serve as a biosensor for the cancer biomarker spermine.	Hasan et al., 2023 [160]
	Lysine-modified β-cyclodextrin (Lys-β-CD)	Lys-β-CD-BER complex may act as a potential sustained-release system, with applications in drug delivery and biomedical fields.	Liu et al., 2023 [162]
Berberine, coptisine, palmatine, epiberberine, dehydrocorydaline	SBE_6_._4_-β-CD	The association constant decreases eightfold in the series berberine ≈ coptisine >> palmatine > epiberberine > dehydrocorydaline.	Miskolczy et al., 2022 [161]
Piperine (PIP)	β-CD methylated derivatives of β-CD HP-β-CD	Complexation efficiency was more elevated using randomly methylated-β-CD and HP-β-CD than using β-CD.	Christoforides et al., 2022 [154]
	β-CD SBE-β-CD HP-β-CD DM-β-CD	PP formed the most stable complexes with SBE-β-CD, followed by HP-β-CD.	Ali et al., 2024 [155]
Mitragynine (MTR)	β-CD SBE-β-CD	Increased water solubility and bioavailability of MTR; MTR forms more stable complexes with SBE-β-CD than with β-CD.	Várnai et al., 2022 [156]
Veratridine (VTD)	β-CD δ-CD SBE-β-CD	VTD formed more stable complexes with γ-CD and SBE-β-CD than with β-CD. Possible use as antidotes for VTD-induced toxicity.	Uribe et al., 2022 [157]
Solasodine (SS)	Sugammadex (SGM) (a modified γ-cyclodextrin)	Possible use as an antidote for SS-induced toxicity.	Kalydi et al., 2024 [163]
Kratom alkaloids	40 CD derivatives (native and synthetic)	Affinity capillary electrophoresis indicated that the highest stability of complexes was achieved for the medium cavity-sized, negatively charged CDs; potential in creating antidotes for kratom and cathinone analogs.	Dohárszky et al., 2024 [164,165]
Cathionine and four of its derivatives			

## Data Availability

All the data are available in the article’s main text. No own experimental data were used for the research described in the manuscript.

## Abbreviations

The following abbreviations are used in this manuscript:

CDs	cyclodextrins
IC	inclusion complex
SMs	secondary metabolites
PPs	polyphenols
Alks	alkaloids
TTs	terpenes and terpenoids
FTIR	Fourier transform infrared spectroscopy
XRD	X-ray diffraction
UV-Vis	ultraviolet–visible spectroscopy
DSC	differential scanning calorimetry
NMR	nuclear magnetic resonance
ESI-MS/MS	electrospray ionization tandem mass spectrometry
CS-GA	gallic acid-grafted chitosan
HP-β-CD	2-hydroxypropylated-β-CD
M-β-CD	methyl-β-CD
DM-β-CD	dimethyl-β-CD
SBE-β-CD	sulfobutyl ether-beta-cyclodextrin
QA-Ch	quaternary ammonium chitosan derivative
